# Supplementary material for: A systematic scoping review of adult obesity policy actions and weight-related services in a region of the United Kingdom using the behaviour change wheel
Source: BMC Public Health. 2026 Jan 29;26:695. doi: 10.1186/s12889-026-26376-7 (PMC12924327; doi:10.1186/s12889-026-26376-7)
Supplement: Supplementary file 1 — Supplementary Material 1 [file 12889_2026_26376_MOESM1_ESM.docx]

Identifying and describing relevant obesity policies/WMS in NI

Search details and adaptions

For the purposes of the current study, four different types of searches were conducted for a comprehensive overview of policy actions/WMS currently in place and that has existed since 2008. Instead of using Custom Google Search engines as in the study by Godin et al., 2015 we replaced this particular search strategy with tailored online searches using “Google Advanced Search”, as was done previously in a recent policy and stakeholder mapping study on social inclusion in the Irish health context (1). Harnessing the power of the Advanced Google search tool using various word combinations and search terms was deemed a more suitable option for capturing all the required information. Original searches were conducted in July/August 2020 and the Google Advanced Searches and targeted website searches were updated in February 2024.

The search plan utilised the search strategies in the below figure:

Figure 1. Search strategies of systematic grey literature search

1. Included Department of Health NI (DoH) website, PHA website, FSA website, SafeFood website, local HSCT websites, Sustrans website, Association for the study of Obesity website, Cancer Focus website, Action Cancer website, NI Chest, Heart and Stroke website, Diabetes UK website, Cancer Research UK website and British Heart Foundation website.

2. Consultation with experts/stakeholders in obesity (Included PHA colleagues, community food and nutrition team/weight management dietitian within a HSCT and SafeFood representative)

Google Advanced Searches

Google Advanced Searches were undertaken as the first step to search for different types of relevant documents and information sources published and/or available online. The search terms and keywords in Table 1 were used to conduct 12 Google searches in July 2020, which were repeated in 2024 to check for updates. As it would not be feasible to screen all results from a Google Search even when using the Advanced Google Search tool (see #results column of Table 1), we screened the first 10 pages (i.e., first 100 results) retrieved from each search, relying on the in-built relevancy ranking of Google as a search engine. The first screen involved reviewing the title and text description to decide if the web link was potentially relevant.

The bookmarking tool on Google Chrome was used to bookmark the potentially relevant records for each search with a folder created in the Bookmark Manager for each set of keywords/search terms. This was a way of recording which websites/links were found through which word combinations before they were exported to an excel spreadsheet. It also served as a means to distinguish between records that had been identified previously and those that were new potentially relevant records. The URL of bookmarked pages are starred with a blue star making it easier to see where a web link has been previously identified. The second screen was of the potentially relevant records identified in the first screen and involved reviewing the entire webpage (or link to a document) to assess eligibility. The number of “hits” and records screened and retained are shown for each set of keywords in Table 1.

Table 1- Details of the Advanced Google Searches

| **#** | **AGS Search** | **# results** | **# results screened** | **# new potentially relevant Weblinks** | **Total # Weblinks** |
| --- | --- | --- | --- | --- | --- |
| 1 | ~obesity AND Northern Ireland OR NI | 19,000,000 | 100 | 20 | 20 |
| 2 | ~obesity Northern Ireland AND policies OR services | 10,800,800 | 100 | 13 | 33 |
| 3 | "weight management" OR "weight loss" AND Northern Ireland | 16,600,000 | 100 | 9 | 42 |
| 4 | obesity prevention OR intervention OR treatment AND Northern Ireland | 10,700,000 | 100 | 12 | 54 |
| 5 | physical activity Northern Ireland AND guidelines OR strategy OR initiative | 178,000,000 | 100 | 25 | 79 |
| 6 | healthy eating OR healthy lifestyle AND Northern Ireland | 59,400,000 | 100 | 18 | 97 |
| 7 | health improvement OR health promotion AND Northern Ireland | 159,000,000 | 100 | 8 | 105 |
| 8 | obesity AND Belfast Health and Social Care Trust 21/08 | 800,000 | 100 | 10 | 115 |
| 9 | obesity AND Northern Health and Social Care Trust 24/08 | 7,460,000 | 100 | 8 | 123 |
| 10 | obesity AND Western Health and Social Care Trust 24/08 | 14,300,000 | 100 | 8 | 131 |
| 11 | obesity AND South Eastern Health and Social Care Trust 24/08 | 14,700,000 | 100 | 4 | 135 |
| 12 | obesity AND Southern Health and Social Care Trust 24/08 | 8,940,000 | 100 | 2 | 137 |
|  | #relevant records after 2nd screen & removal of duplicates? | | | | 53 |

Grey literature databases

The second search strategy entailed searching relevant grey literature databases for obesity policy documents (or similar) relevant to NI (in August, 2020). Searches of three databases were carried out, namely the “RIAN-Pathways to Irish Research” database, NHS Evidence and Open Grey using the keywords/search terms “obesity AND Northern Ireland”. The results from each database search were exported to an excel file, screened by title and then further screened a second time, before duplicates were removed. The grey literature searches were not repeated in 2024 as two of the main databases (RIAN and NHS Evidence) were not in operation any longer. In addition, it was felt that there were no new policy documents of relevance to obesity in NI available since the last search, considering the new Healthy Futures Obesity Strategy for NI was still in consultation.

Targeted website searches

This involved hand searching and browsing *n=17* websites of relevant health/health promotion agencies, HSCTs, charities and non-governmental organisations (in August 2020 and updated in February 2024). If available, the search function embedded within the website itself was also used. These websites were selected based on recommendations derived from the expert knowledge of the research team and by gauging which organisations repeatedly featured in the Google Searches (publishing relevant material to the subject of interest).

Stakeholder consultations

In order to verify what had been found in the various searches and to identify any additional potentially relevant records (policy actions/WMS), stakeholders with expertise in obesity in NI were contacted. This resulted in consultations with representatives from the PHA, SafeFood and a Southern Trust dietitian who is a member of the NI British Dietetic Association Board with specialist knowledge of obesity (completed by December 2020). Ethical approval was not required for this element of the study as it focused only on verifying the findings relating to policies/WMS for each relevant organisation. Formally repeating stakeholder consultations as part of the 2024 update was not deemed necessary, due to adequate retrieval of updated policies/WMS from other search updates.

1. O’Donnell P, O’Donovan D, Elmusharaf K. Social inclusion in the Irish health context: Policy and stakeholder mapping. Irish Journal of Medical Science (1971 -). 2020;189(1):11-26.
