# Supplementary figures and images for: A systematic scoping review of adult obesity policy actions and weight-related services in a region of the United Kingdom using the behaviour change wheel
Source: BMC Public Health. 2026 Jan 29;26:695. doi: 10.1186/s12889-026-26376-7 (PMC12924327; doi:10.1186/s12889-026-26376-7)

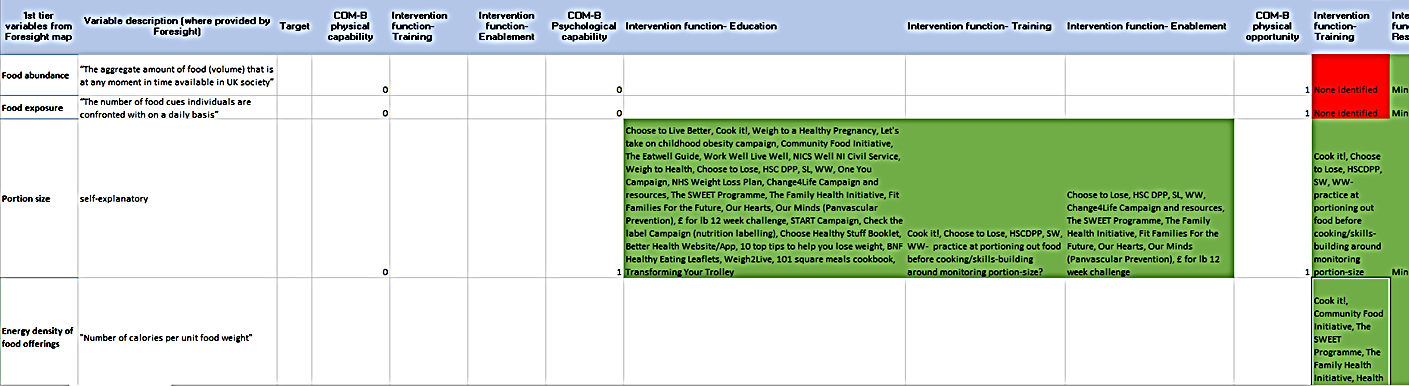


Example snapshot of cross-reference grid which informed the creation of the heat map

Supplement: Supplementary file 5 — Supplementary Material 5 [file 12889_2026_26376_MOESM5_ESM.docx]
