# Supplementary material for: A systematic scoping review of adult obesity policy actions and weight-related services in a region of the United Kingdom using the behaviour change wheel
Source: BMC Public Health. 2026 Jan 29;26:695. doi: 10.1186/s12889-026-26376-7 (PMC12924327; doi:10.1186/s12889-026-26376-7)
Supplement: Supplementary file 6 — Supplementary Material 6 [file 12889_2026_26376_MOESM6_ESM.docx]

| **Behaviour Change Wheel Intervention type** | **Number of mapped policies out of possible opportunities** | **% coverage** |
| --- | --- | --- |
| **Education:** Increasing knowledge or understanding | 25/29 | 86† |
| **Persuasion:** Using communication to induce positive or negative feelings or stimulate action | 22/37 | 59 |
| **Incentivisation:** Creating an expectation of reward | 4/37 | 11* |
| **Coercion:** Creating an expectation of punishment or cost | 1/37 | 3* |
| **Training:** Imparting skills | 22/55 | 40 |
| **Restriction:** Using rules to reduce the opportunity to engage in the target behaviour (or to increase the target behaviour by reducing the opportunity to engage in competing behaviours) | 10/38 | 26* |
| **Environmental restructuring:** Changing the physical or social context | 41/56 | 73 |
| **Modelling:** Providing an example for people to aspire to or imitate | 21/38 | 55 |
| **Enablement:** Increasing means/reducing barriers to increase capability (beyond education and training) or opportunity (beyond environmental restructuring) | 48/75 | 64 |

Table 4- Policies/WMS in place by Behaviour Change Wheel intervention type (scored out of possible opportunities)

*Indicates those less than 40%, † Indicates those over 80%
